# Draft Genome Sequence of the Feruloyl Esterase-Producing Strain Lactobacillus fermentum CRL1446, a Probiotic for Malnutrition

**DOI:** 10.1128/genomeA.00225-18

**Published:** 2018-05-24

**Authors:** María C. Abeijón Mukdsi, Lucila Saavedra, María P. Gauffin Cano, Elvira M. Hebert, Roxana B. Medina

**Affiliations:** a Centro de Referencia para Lactobacilos (CERELA)–CONICET, San Miguel de Tucumán, Tucumán, Argentina

## Abstract

We report here the draft genome sequence of Lactobacillus fermentum CRL1446 (2,148,781 bp, 51.4% G+C content). This strain exhibits feruloyl esterase activity and important technological and probiotic properties. Because of its proven beneficial effects *in vivo*, it represents an interesting candidate for the development of functional foods or pharmabiotics for malnutrition.

## GENOME ANNOUNCEMENT

Lactobacillus fermentum CRL1446 is a strain isolated from an artisanal goat milk cheese produced in northwest Argentina. It presents optimal technological properties for the development of fermented dairy products, mainly cheeses, due to its capacity to generate flavor compounds (1). Furthermore, this strain displays probiotic properties and feruloyl esterase (FE) activity, which is capable of releasing antioxidant ferulic acid from its nonbioavailable esterified forms naturally present in vegetable foods (2). Free ferulic acid can be absorbed in the gut and can exert several health benefits on the host (3).

The administration of L. fermentum CRL1446 to mice subjected to normal and malnutrition diets (caloric restriction and high-fat diets) increased intestinal FE activity and enhanced oxidative status. Moreover, it reduced glycemia, cholesterolemia, and triglyceridemia and positively modulated intestinal microbiota, as evidenced by a marked bifidogenic effect (4–7). This noninflammatory strain, which is able to modulate the profile of adipokines *in vitro* (8), also reduced body weight gain and leptin levels in diet-induced obese mice (7). Due to these functional properties, L. fermentum CRL1446 could be used as a probiotic for reverting or improving alterations related to malnutrition.

Here, we present a draft genome sequence of L. fermentum CRL1446 that was sequenced using a whole-genome shotgun strategy on an Illumina HiSeq 2500 sequencer. Paired reads with lengths of 250 bp were obtained, corresponding to a 670-fold coverage. Quality-filtered reads were assembled using the NGEN assembler (DNAStar version 12.2.0), resulting in 56 contigs. The Rapid Annotations using Subsystems Technology (RAST) server and the NCBI Prokaryotic Genome Annotation Pipeline (PGAP) were used for functional annotation of predicted genes (9).

The 2,148,781-bp genome is composed of a single circular chromosome with a G+C content of 51.4%. The chromosome contains 2,187 coding sequences and 74 RNA genes, as predicted by PGAP (15 rRNAs, 56 tRNAs, and 3 noncoding RNA genes). There are 316 subsystems represented in the chromosome, which comprise only 49% of the sequences assigned. According to the RAST analysis, 1,599 protein-encoding genes were assigned to known functions.

*In silico* studies revealed that L. fermentum CRL1446 carries eight genes encoding proteins predicted as esterases/lipases (E.C.3.1.1.x), which belong to the α/β fold hydrolase family and display the classical serine nucleophilic motif (GxSxG) described for some carboxyl esterases (10). One of these predicted proteins (GenBank accession number PNV58462) shares 100% identity with a FE from L. fermentum SD1210 (GenBank accession number AOR52356).

Further in-depth analysis of the L. fermentum CRL1446 genome will help to exploit the biotechnological and probiotic potential of this strain and elucidate the mechanisms underlying the beneficial effects observed *in vivo*. This information could support the use of L. fermentum CRL1446 for developing novel functional foods, dietary supplements, or pharmabiotics directed to malnutrition and other oxidative stress-related ailments.

### Accession number(s).

This whole-genome shotgun project has been deposited at DDBJ/EMBL/GenBank under the accession number POTQ00000000. The version described in this paper is the first version, POTQ01000000.
